# “Sharing Is Caring:” Australian Self-Trackers' Concepts and Practices of Personal Data Sharing and Privacy

**DOI:** 10.3389/fdgth.2021.649275

**Published:** 2021-02-23

**Authors:** Deborah Lupton

**Affiliations:** Vitalities Lab, Centre for Social Research in Health and Social Policy Research Centre, University of New South Wales (UNSW) Sydney, Kensington, NSW, Australia

**Keywords:** self-tracking, Australia, data sharing, social research, interviews, relational, more-than-digital, data privacy

## Abstract

Self-tracking technologies and practices offer ways of generating vast reams of personal details, raising questions about how these data are revealed or exposed to others. In this article, I report on findings from an interview-based study of long-term Australian self-trackers who were collecting and reviewing personal information about their bodies and other aspects of their everyday lives. The discussion focuses on the participants' understandings and practices related to sharing their personal data and to data privacy. The contextual elements of self-tracked sharing and privacy concerns were evident in the participants' accounts and were strongly related to ideas about why and how these details should be accessed by others. Sharing personal information from self-tracking was largely viewed as an intimate social experience. The value of self-tracked data to contribute to close face-to-face relationships was recognized and related aspects of social privacy were identified. However, most participants did not consider the possibilities that their personal information could be distributed well-beyond these relationships by third parties for commercial purposes (or what has been termed “institutional privacy”). These findings contribute to a more-than-digital approach to personal data sharing and privacy practices that recognizes the interplay between digital and non-digital practices and contexts. They also highlight the relational and social dimensions of self-tracking and concepts of data privacy.

## Introduction

Self-tracking as a practice of generating details about individuals has also attracted much attention in recent years, particularly in relation to the use of digital media and devices. A multitude of digital technologies are now available for people to engage in monitoring aspects of their bodies and everyday activities (1, 2). Spreadsheet software, apps, wearable devices and platforms are among the technologies that can facilitate the recording, storage and analysis of personal details. The personal data that are generated from digitized modes of self-tracking include details of trackers' physical location and movements in space, biometrics such as body weight, physical activity, heart rate, mood, sleep patterns, alcohol and other drug use, sexual behavior, intimate relationships, sexuality, reproduction, and fertility cycles. Other aspects of people's everyday lives can also be monitored, including finances and relationships energy use.

The sharing ethos has become a central aspect of online interactions. Across many social media platforms and online discussion forums, users are encouraged to upload details for the perusal of other users, sometimes involving commenting or re-sharing of this content. Online sharing can lead to feelings of community, social engagement, intimacy and support (3), but it also has potentially negative consequences. People may be considered by others to go too far in self-disclosure of sensitive or overly mundane details about themselves: or to “overshare” (4). Further, once these personal details go online, they may become public and difficult to erase, or become open to exploitation by third parties in often unknown ways (5). A tension therefore exists between the sharing ethos of digital media and concerns about the privacy of the personal details that are exposed online (6).

People who monitor and record aspects of their lives are often encouraged to share their data online. For example, the “show-and-tell” mode of performing self-tracking and publicly revealing personal details is a standard practice for members of the Quantified Self community, a worldwide network of groups of people who connect on the Quantified Self website and organize meetings (1, 7). This practice involves attending meetings of members or the annual Quantified Self conferences and delivering a presentation that focuses on how the presenters practice self-tracking and what insights they have gained from it. Some of these show-and-tells are video-recorded and uploaded to the official Quantified Self website and thus made available to any internet user as online information resources (7, 8). Sharing of personal details is also frequently promoted on some weight-loss and fitness-tracking platforms (9–13). Content creation and sharing platforms such as YouTube, Instagram and TikTok provide further opportunities for people to upload and share self-made images portraying their experiences of health and embodiment (14–17).

Online patient support groups such as PatientsLikeMe as well as Facebook groups and other social media also encourage members to share their health, fitness and medical details as a way of contributing to peer networks of expertise and support (18–22). The athletic monitoring platform Strava refers to the sharing of details about their physical activities by its members as “social sharing:” a way of both receiving and bestowing support and encouragement between members (11). Other forums can take on a confessional tone, where users reveal “bad” data and seek redemption and moral support from other users (13, 20, 23).

The sharing economy, however, is also serving the demands of potentially exploitative data economies. The expansion of opportunities for digitized modes of self-tracking has brought potential risks and harms concerning how trackers' personal details may be accessed and exploited by third parties in unprecedented ways. The use of sensitive personal data by data companies for profiling, sorting and prediction is increasingly common (24). These digital data can be harvested and on-sold for profit by the companies that offer the devices and software for self-tracking (10, 25). Health and medical data are particularly valuable in the digital data economy for both legal and illicit purposes (7, 21, 26). Data collected by health and fitness-tracking apps and wearable devices may be inadvertently breached or leaked, or hackers and cybercriminals may deliberately access the data illegally (27). Privacy advocates and social researchers have consequently become increasingly concerned about the ways in which the personal data generated from digitized self-tracking and other modes of engagements with apps and online technologies can be used to exploit people, deny them opportunities or otherwise exacerbate their social disadvantage and marginalization (10, 28–31).

In this article, I report on findings from an interview-based study of Australians who have actively engaged in long-term self-tracking aspects of their bodies and lives. In Australia, many people are now encouraged or required to engage in self-tracking in the interests of their health, physical fitness or productivity. Customer loyalty schemes offered by major retailers, including the popular “Flybuys” program, encourage Australians to use fitness trackers to earn points (32). As is the case in other countries, Australians who generate personal information about their everyday activities using online or mobile technologies are open to potential misuse of their data (31). Furthermore, several highly publicized personal data breaches and cases of government misuse have occurred in Australia in recent years (33, 34).

The present discussion focuses on two related issues concerning the personal information generated by the participants by way of their self-tracking practices: their data sharing and their privacy understandings and practices. This study contributes to the literature on self-tracking, personal data sharing and privacy by investigating the perspectives of Australians from diverse ages and backgrounds who identify as self-trackers, monitor not just health-related but other aspects of their lives and use a range of methods, including non-digital. This is a sociodemographic group and range of practices that hitherto have not received much previous attention from social researchers. Several interview-based studies by sociologists and anthropologists have been published that include Australian self-trackers (35–42). These studies have surfaced intriguing dimensions of Australians' feelings about and experiences of self-tracking and sense-making in response to their personal data, but thus far have not devoted extended attention to participants' attitudes and practices relating to personal data sharing and privacy.

I begin with a background section that provides discussion of previous related empirical research. My empirical study of Australian self-trackers is then described, followed by a discussion of findings from the research and their implications for understanding practices and understandings related to self-tracked data sharing and privacy. The findings emphasize the interpersonal, relational and affective dimensions of these practices. The analysis and discussion, therefore, work toward developing concepts of personal data sharing and privacy that acknowledge the importance of both digital and non-digital dimensions.

## Self-Tracking Data Sharing and Privacy: Previous Research

Social research has identified a range of practices and feelings concerning sharing self-tracked information with others. Studies have found that keen athletes who use “social fitness” platforms such as Strava often appreciate the support, competition and feelings of achievement and prowess that they experience with uploading their physical activity details to the platforms for other members to view and comment on (9, 10, 12). Some self-trackers make distinctions between modes of sharing personal information. German self-trackers were found to identify “pragmatists,” who tracked for health or weight-loss reasons, and rarely revealed their data to anyone, and “enthusiasts,” who enjoy self-tracking for itself and are more likely to share their data with other self-tracking app users (43). Focus group discussions including English school students who had been given Fitbit wearable devices to track their physical activity (44) revealed that they felt ambivalent about sharing their data with their peers. Some found it motivating and enjoyed competing against their friends, while others stopped sharing because they did not enjoy the competitive elements.

Concepts of privacy have been reassessed in the light of the voluntary sharing of information that occurs on online platforms. Instead of the traditional westernized theory of “autonomous privacy,” which represents it as related to the rights of individualized selfhood (45), privacy scholars now frequently argue for a “networked privacy” approach. This perspective acknowledges that sharing of personal details online often inadvertently involves revealing other people's details. Therefore, privacy in relation to online data cannot be understood as the right or privilege of an individual (46). Another concept that has been offered in response to new digital media communication is that of “relational privacy” (45). Both the networked privacy and relational privacy concepts critique the idea of autonomous privacy, highlighting the distributed nature of privacy as it is performed in digital media. The concept of “contextual privacy” also acknowledges that the relational, spatial and temporary aspects of personal data sharing shape privacy concepts (47). Another distinction has been drawn between “social privacy” and “institutional privacy” in terms of how social media users understand privacy. Social privacy refers to the extent to which known intimate others, such as friends and family, can access and view personal information uploaded online. Institutional privacy concerns how institutions such as corporations and government agencies access this kind of information (48, 49).

A body of literature has developed using qualitative methods to investigate how publics understand and practice online data sharing and privacy in relation to their social media use [for example, (50–54)]. Thus, far, however, only a small number of studies have focused specifically on self-trackers' attitudes and practices related to the privacy and security of the personal details they choose to generate about themselves, whether online or face-to-face. One project involving interviews with Danish self-trackers identified that few of them used dedicated self-tracking apps or platforms to communicate with other people engaging in self-tracking and that they had not devoted much thought to third-party use of their data (55). Another study used a survey and semi-structured interviews with fitness tracker users employed in two American universities (56). The interviewees expressed few privacy concerns about what happened to the information generated by their fitness tracking device. They could not readily envisage ways in which third parties might be interested in their fitness data and considered their data well-protected. A Canadian project involving an online survey with users of wearable devices for self-tracking their fitness activities (57) similarly found that the participants expressed little knowledge about data privacy and lacked interest or concern in protecting their personal information from others' use or exploitation.

In a British online survey administered to people who tracked their food intake and physical activity, including those with Irritable Bowel Syndrome and diabetes (58), once again, respondents demonstrated lack of understanding about the potential reuse and sharing of their data. Another British study involved interviews with people who identified with the Quantified Self community (7). It found that these committed self-trackers believed that sharing their information with other members could be beneficial and enjoyed doing so as a social activity but did not consider the implications of anonymous third parties accessing it. They could see little value in their personal data for others beyond community sharing and support purposes.

As noted earlier, research on how Australians who generate details about themselves share this information and their views and practices related to their data privacy is limited. In what follows, I build on and extend the findings from these previous studies in the UK, North America and Europe by presenting an analysis of interviews with Australians who identify as self-trackers and focusing on what they said about how they shared their data and data privacy issues. In the discussion section, I return to concepts of data privacy, building on those of networked and relational privacy to advance a perspective that acknowledges personal data sharing and privacy practices as occurring in non-digital contexts.

## Materials and Methods

When designing the Australian Self-Trackers project, I was interested in asking people who were currently self-tracking using any method about their rationales and lived experiences of monitoring aspects of their lives: essentially addressing the questions of “how” and “why” people engage in these practices. Forty participants from around Australia who engaged in some form of self-tracking took part in a qualitative semi-structured telephone interview in mid-2016. The study was approved by the University of Canberra human ethics committee (I was affiliated with that university at the time). The participants were drawn from people who had volunteered to join the panels of a major Australian research company. Panel members were recruited by the company, who sent out an invitation to participants aged 18 years and over with the screening question: Do you self-track any aspect of your life? This question was deliberately general, as I wanted to see how people interpreted the concept of self-tracking and applied it to their lives.

Those people from the panels who responded to the invitation to participate were provided with the participant information details and consent forms by email and gave their consent by return email once they had reviewed the information. A time was then organized to conduct their interview by telephone. The interview schedule I developed included questions about what people were self-tracking, the methods they were using, why they took up self-tracking, what benefits they gained from it, any frustrations or disappointments they had experienced, how accurate they thought the information they gathered was, whether they shared it with anyone else and whether they had any concerns about the privacy or security of their personal data. The interviews took an average of 30 min to complete. Participants were provided with a gift card worth AU$50 as a token of appreciation for completing the interview. They were all given pseudonyms to protect their anonymity.

The participant group was deliberately recruited to be diverse in terms of gender, age, area of residence and education levels. To ensure an even gender and age spread, sub-quotas were set of 20 men and 20 women, 20 participants aged 40 or under and 20 aged over 40 (the oldest participant was aged 75). The vast majority of participants who agreed to participate resided in the most populous states of Victoria (45 per cent) and New South Wales (48 per cent), and in the capital cities of those states: 80 percent of the participants came from either Sydney or Melbourne (the cities that together are home to 40% of Australians), with the others hailing from Adelaide, Brisbane or regional cities in New South Wales or Victoria. Half held a university undergraduate or postgraduate degree (53 per cent) while the remainder had high school qualifications.

The interviews were transcribed, and I used these transcripts for analysis. I was particularly interested in surfacing the participants' accounts of the affective forces and relational connections (38) that were part of their practices and understandings, and the situated nature of these elements. How did the participants feel about sharing and data privacy? How did their practices contribute to their social relationships? Elsewhere (59, 60), I have discussed what aspects of their lives the participants were tracking, how they achieved this regular generation of personal details, and the rationales they gave for self-tracking. The participants were mostly tracking intimate aspects of their lives and bodies such as their health status, physical activity and food intake. Several people were also tracking aspects such as their finances, work productivity, social relationships and home energy use. Nearly all the participants were using digital technologies to generate, store and present their personal information, albeit often in concert with non-digital methods such as using pen-and-paper. They recounted the importance of self-tracking for helping them to establish better knowledge of their bodies and their lives, identify patterns in their health or activities, set and reach goals, feel more in control of their health and aspects such as their finances, solve problems and feel or function better and more. In what follows, I focus on the participants' responses to the questions they were asked about whether they shared their self-tracked data with anyone else, and whether they had any concerns about the privacy or security of their personal data.

## Findings

### Sharing Personal Data

In recent years, great prominence has been given to the Quantified Self terminology and community in media coverage as well as by academic researchers (1). However, none of the participants identified as members of the Quantified Self community, made any reference to the quantified self or referred to themselves as engaging in “quantifying” themselves. Nor did these participants fit the stereotype of the self-obsessed self-tracker who is narcissistic and overly enthusiastic about communicating personal details of their lives with others as a form of self-promotion and aggrandizement (1).

The practice of online data sharing was rare among this group of self-trackers. Very few participants said that they shared their personal data with other people using online platforms or social media. When explaining why they did not engage in online sharing, for the most part, the participants said that they didn't see the point, as they thought about self-tracking as a personal and private matter, something they did only for themselves:

I think it's just a personal thing. It's like a motivation thing for me really to be healthy, so I don't really want to share that with other people, I guess (Andy, aged 31, wholesales marketer).

Roger is a 50-year-old project manager who tracks his finances and many aspects of his health: his body weight, physical fitness, sleep and blood pressure. He emphasized that he chose not to share his self-tracking data online because he did not identify as “one of these social media people” and therefore “I don't really feel the need to share that I've walked this many kilometers or something like that.” His comments demonstrate the negative connotations that over/sharing on social media can have among people who monitor themselves.

Not all participants were anti-sharing. Instead, they were selective about who they shared with and how. Jason, a 29-year-old teacher, commented that he occasionally shares his running data on Facebook, but usually he simply talks about the data with his life partner or with friends who share his interest in running. Jason noted that he engages in sharing for two reasons: either to receive acknowledgment for good results, or support and advice if his results are not as favorable:

If I've done a few personal bests of running, if I've acquired more points from my Moves app that week—yeah, I'll share it with my friends or put it on Facebook. Sometimes I'll tell my partner if I've had a bad week. I guess if it's an activity and it's a positive result, I guess I'm showing off my ability at running. If it's a negative result, if I did badly or the data shows something, then I'm showing my friends because I want them to reply to me with solutions.

When people did share their self-tracked details with others, this tended to be part of an established face-to-face relationship. Some participants gave examples of letting friends, family and work colleagues know about how best to improve one's health, fitness or financial situation by drawing on their self-tracking experiences and knowledges. Dan (aged 29, project manager) said that he talked about his self-tracking data about his exercise with his life partner. In his case, it was to provide encouragement for her to engage in a similar self-tracking activity by demonstrating the value of the app he was using.

I just share with my partner, nothing online. Mainly because she's interested in doing the same thing. So the app's quite cool with showing you graphs. So I'm trying to get her on board by showing her some of the cool functions of it.

Maria (aged 40, home duties) tracks her finances and health details such as food intake and body weight. She had grown up experiencing significant financial difficulties and said that she was happy to share her financial self-tracking acumen with close family members as a way of helping them protect themselves against debt. She was particularly keen that her adult children learn these skills as part of becoming independent.

I share with friends and family, so that it can help them whenever they choose to lose a bit of weight or something. Or so that they learn how to manage their finances or handle their asthma problems, or whatever.

Kerry (aged 61, youth worker) shared what she had learned from monitoring her energy consumption with her friends to help them save money. Amy, a 29-year-old clerical worker, noted that she sometimes talks about her self-tracked information with close friends and family as a way of empathizing with and supporting them:

Just to like, get views and if they are suffering from pain, things that I've got to know about it—what I have to do. And how is it feeling when someone else is also having the same problem—that's why I'm sharing with them.

Some participants said that they had mutual goals with their partners, and therefore pooled information as part of working toward these goals. Thus, for example, both Chloe (aged 23, recruiter) and Vanessa (aged 29, science industry) reported that they shared their financial tracking information with their partners. As Chloe noted: “It's just easy, he's my partner and it's something that we do together”.

Rob, aged 52 (self-employed), provided a detailed explanation of the rationale behind his decisions about sharing his self-tracked information. Rob said that his main self-tracking activities centered on his finances and health details such as food and alcohol consumption, exercise, sleep and body weight. He had successfully lost weight in recent times and attributed his self-tracking to helping him achieve this goal, as well as drawing people's attention to his practices because it was such a visible outcome.

Depends on who is the audience. Some details I do reveal, finances and other things within the family. My wife will know about those sorts of things. What I collect daily I share it with a couple of my friends, but apart from that no. I keep my tracking private. Some of the charts are available online. But for financial matters I also do share with my son. As I say it depends on the audience. Physically I've changed a lot and so people might ask for details. Financial details I don't want to share because it's a private matter and things like that.

In these participants' accounts, sharing tended to be considered an act of altruism, a way of reaching shared goals, sharing insights that have been learned through self-tracking or a way of disclosing life experiences as part of building on personal intimacy with friends and family. Howard's view on the importance of sharing self-tracked details with intimate others insightfully sums up most people's attitudes. As Howard noted, “sharing is caring:” it is a way of exchanging personal details that strengthens social bonds:

I occasionally share my details, yes. Not online, but face-to-face. Family members and friends. I think it's because there's a value in sharing stuff with people rather than not, if you know what I mean. Somehow, it's like if you share that secret, it's a part of bringing people into your personal life. Sharing leads to caring in that way, I guess.

For a smaller number of participants, sharing self-tracked data was part of their relationships with expert advisors who helped them with achieving their goals. Several people shared financial details with their financial planners as part of management plans. People with chronic health conditions that they were monitoring also often shared their information with their healthcare providers, frequently at the suggestion or request of the providers. For example, while Roger was loath to share his health details online, he did note that he shared them with his doctor during consultations. David, a 61-year-old teacher, is another example. He lives with diabetes, requiring high levels of daily self-monitoring to keep the condition under control, as well as attempting to manage his sleep apnoea. David said that he provides regular updates of his self-tracked details to his general practitioner and medical specialist when his attends appointments. He also often chats to the nurse at his workplace and provides her with details about his conditions. David said that he finds these conversations supportive and encouraging, and he enjoys discussing his health details with her.

Greg, a 57-year-old workplace trainer, who is managing high blood pressure and trying to control his weight. He said that sharing his biometrics with his doctor is important as part of his regular medical checks:

At the moment I show my details to my GP because he has insisted, particularly with my hypertension, that I self-track. And I visit him every 6 weeks, and he wants a weekly average of my pulse rate, blood pressure rate and so on and I give him that. And he can determine then if the medication I'm on is sufficient or whether it's too much, and whether it should be reduced or increased. So that's the advantage of it, by keeping a record, particularly with my weight as well.

For these participants, health professionals were portrayed as expert partners in self-tracking efforts, providing important feedback and advice on the information the self-trackers collected about themselves. In this representation of self-tracked data, people viewed these experts as the arbiters and assessors of the meanings of the data.

In summary, for these long-term self-trackers, sharing their personal information was a highly situated and contextual practice. People considered aspects such as what kind of information they had collected and with whom it was most appropriate to share it when deciding whether they wanted to allow other people access to it and felt comfortable about doing so. What was particularly evident in their decision-making were aspects such as how close a personal relationship was, how sensitive or revealing the data were, whether the self-tracker saw themselves as able to help or instruct others by sharing their information or whether the recipient of the information was a trusted expert who had requested the data as part of helping the person achieve goals such as managing their health condition or improving their financial situation.

### Personal Data Privacy and Security

Even though the participants held firm views about how and with whom they should share their self-tracked details, few had given much consideration to how third parties such as software developers, government agencies or hackers might seek to access and use their data. The potential for their data to be exploited by third parties and the possibilities of data leakages, breaches or hacking did not seem to have entered their horizons. As Ian, a 41-year-old engineer, described his perspective in relation to downloading and using the cycling monitoring app he used:

I haven't really thought about it. When I installed the app, I just accepted terms and didn't pay much attention. I suppose they could track where you are at any time. I hadn't really worried about it.

In many cases, participants simply did not think that their personal information would be of interest or use to anyone else. Several people's responses suggest that some self-tracked details are considered to require more protection from access by third parties than others, because they reveal more private or sensitive information about the person. These participants felt that because they were not tracking these details, they were not concerned about other aspects of their lives that were being recorded by their apps or other self-tracking software. Physical activity and fitness details, for example, were considered to be less sensitive or revealing than data about people's finances, sexual or reproductive functions, or emotions.

At the moment I'm not bothered about where stuff goes. If they were tracking my feelings or emotions, then I probably would check. But because I'm mainly tracking work I don't really care (Emma, aged 18, student).

No, I'm not really concerned about other people seeing my data. As long as it's not too personal. I wouldn't do fertility or sexual tracking apps. But things like running, as long as it's not too personal to me then I don't really mind. I doubt they'd ever care enough (Jason).

When participants did express a degree of concern about how well their self-tracked information was protected, this was often in relation to their awareness that advertising companies could access their data from online interactions. As Carol (aged 57, retired teacher) noted:

I'm just a little bit concerned. Sometimes with the computer, I know that they're using my knowledge or information to try and pass it back onto me through ads. They do know I'm looking at trying to purchase things.

For Sharon (aged 43, self-employed), access of people's personal data was an inevitable and unpreventable outcome of using online services. Furthermore, she thought that data companies were not able to access aspects of her identity that she considered important to protect.

No, I don't think anyone cares about that. That's long gone, caring about things like that. The minute you use the internet you're already open to it all. And also, they don't actually care about me, they care about just that person who fits those statistics. Generally speaking, apps and websites are tracking to get ad sales, they're not tracking me to find out my identity.

Some people said that they had considered the risks to some extent but were still not very concerned. They had accepted the possibility that their data may be breached or hacked but did not see it as a major threat. As James (aged 39, consultant) observed, to his knowledge, he had not experienced any data breaches, so he remained confident that his data were well-protected by the companies he used:

In general, all applications I log into to provide the information, I believe they maintain that as confidential. Probably they use it for research purposes, but I don't think my personal details are out for anyone. I haven't encountered so far, any breach or been put in an uncomfortable situation. Most of the companies do work in maintaining it.

Rob said that he was careful about sharing personal details online for the very reason that he can then know who can see his information. He feels that because he is careful, he is therefore not open to personal data breaches.

Yes it's always important about what you put on the internet and social media. I make sure that people will not be able to track me down. I try to detach my identity and what else is on, I think I'm in control of that data and information myself.

For Maria, the benefits of generating personal information and sharing it with others far outweighed any potential negative repercussions:

I'm not worried, because I know a lot of the self-tracking stuff I'm doing comes with positive results. And I just like to share those positive results with others so that they can have positive results.

Michael (aged 35) and Matt both worked in IT and were therefore more aware than most of the other participants about personal data security risks. Michael said that he was wary of using apps for storing his financial information or health-monitoring if they asked for details such as his email address, largely because he did not want to be targeted for advertising purposes. Matt was the only person in the participant group to raise the potential of his personal information becoming de-identified and used by agencies such as health insurance companies to discriminate against him because of his health problems:

Of course, I'm concerned that it's maybe going to get out. I know apps have a lot of data and use my data but if I'm a dot point somewhere I don't really mind. What I do have an issue with is if I'm identifiable and they get my weight data, or an insurance company got data from my Fitbit and say Fitbit told me you were this weight at this time and therefore I'm not going to let you claim, that I have a problem with. It's really when they use it against you and you specifically get identified, I have a problem with that.

Not only did the majority of participants express few concerns about third-party use of their self-tracked data, when they were asked whether they would continue to engage in self-tracking into the future, they also often imagined idealized future scenarios in which ever-more personal information would be generated about their bodies, health and activities. Thus, for example, Nick sees himself as tracking even more in the future, as more apps are developed:

More and more apps are being built, and more are generally being useful, and some apps will probably integrate different sections. Maybe in the future we'll have one app which will cover my health, my finance, and have everything under one umbrella.

Roger was also enthused about the idea of future self-tracking technologies that would be even “smarter” and more convenient to use:

I assume the accuracy of technology is only going to get smarter. What I would like to see is taking pictures of people's food on your phone and working out what it is! That'd be great where you just have a system where you just scan a number and work out what to order, for tracking diet. It'll come along with the technology they make. I assume that at some point when you go to the doctor's you'll be able to scan and it records the information from the visit.

Far from expressing ideas about digital technologies that could better protect privacy, these imaginaries often centered on the idea that digital technologies would be better customized and personalized to their users. For committed self-trackers, convenience and time-saving developments in self-tracking technologies trump concerns that more detailed monitoring might lead to even greater opportunities for third parties to exploit or access these data.

## Discussion

In directing attention to how self-trackers conceptualize and practice personal data sharing and privacy, this study has taken an approach that builds on and extend previous work on self-tracked data privacy and sharing by identifying the importance of intimacy, relationality and the non-digital ways in which people respond to and share their personal details. Most previous studies have focused on people's choices about sharing their self-tracked details online. I have shown the complexity of self-trackers' ideas about how their data should be shared, identifying the ways that self-trackers' use of their personal details responds to and strengthens their connections to people with whom they have close personal relationships that are mostly in face-to-face contexts.

While the sharing of self-tracked data is championed in public-facing discourses on self-tracking—and particularly by the Quantified Self community—the technological affordances offered by apps and platforms to easily share personal data and invite the responses of other users were resisted or ignored by nearly all the members of this group of self-trackers. Very few of the participants engaged in online sharing or broadcasting practices with their personal information. Instead, they chose to keep their personal details largely to themselves or discussed them in encounters with significant others in their lives, such as their partners, other family members, or else with experts with whom they already had an in-person relationship, such as their healthcare providers or financial advisors. The potential capacities for sharing offered by the affordances of these technologies, therefore, were largely not taken up in the participants' enactments of self-tracking: not because of their concerns about data privacy, but due to the participants not envisaging any purpose or benefit for this kind of sharing.

These findings highlight the more-than-digital aspects of data sharing, with implications for concepts of data privacy. They expand the concept of “social privacy” (48) by showing that self-tracking data can be used for performances of relationality in social contexts that lie outside online communities or networks. The original concept of social privacy referred to people's views and decisions about sharing their personal information with known others online (48). My findings highlight the affective forces and relational connections that can be part of a concept of social privacy which relates to how self-tracked information is shared offline with others who are known well to people or who hold key roles as expert advisors. Affective forces such as the desire to build and strengthen intimate in-person relationships and to demonstrate support, interest in and care for others were central to the participants' logics of sharing and privacy.

The contextual elements (43, 47) of self-tracked sharing and privacy concerns were evident in the participants' accounts and were strongly related to ideas about why and how these details should be accessed by others. As expressed in the participants' accounts, these elements included ideas about the purposes of self-tracking, who should be given access to the personal details that people collect and how sensitive or vulnerable to exploitation were the details they collected about themselves. In these considerations, we see personal data privacy to be understood as “social” in terms of evaluating what value these details have for contributing to intimate relationships. This is a different concern from people worrying about “institutional privacy” (48, 49), or that third parties might exploit their data without their knowledge or consent. Most of the participants did not define their personal details as vulnerable to institutional privacy threats and thus did not position or frame their sharing and privacy practices in response to this idea of privacy. Unlike people with highly sensitive health and medical data stored online, such as details of sexual practices and HIV status (31, 49, 61), these participants did not view their data as sensitive or stigmatizing, but rather as banal and boring, and of little possible interest to any third parties beyond their immediate social circles or to experts with whom they already had shared goals.

For these participants therefore, self-tracked details were not simply viewed as “information” for the optimisation of lives and bodies or ways of communicating with oneself or others who are interested in self-tracking (62). They are also affective and relational resources. These personal details were considered helpful to communicate with these intimate others as part of advising and supporting them. But practices of sharing these data also had value for the participants as a way of demonstrating their care for and interest in others and as topics for conversations in which every day experiences were exchanged. These kinds of personal data sharing, similar to those recounted by German (43) and Danish self-trackers (55), were less about the actual information and more about conforming to affects, social norms and expectations related to close interpersonal relationships. These findings suggest that data sharing in these contexts work together with other self-disclosing communicative acts that are central to close or expert relationships. The affective forces and relational connections highlighted in these accounts contributed to maintaining the intimacy and reciprocity of familial and family connections, and in some cases, supporting relationships with experts who were partners in helping people to better manage financial matters or their health. Particularly for people who were dealing with chronic health conditions, sharing their self-tracked data gave them the opportunity to contribute to the therapeutic relationship they had with their healthcare providers, generating trust and security.

It is notable that despite the participants acknowledging the highly relational and intimate dimensions of self-tracked data sharing, their concepts of data privacy tended to conform to the notion of autonomous privacy, rather than viewing privacy as networked or relational (45). They expressed a high level of faith in the security of the personal information they generated with the use of digital technologies, which potentially are at much greater risk of access by unknown others compared with details they record with pen-and-paper. Similar to findings conducted in other high-income countries (7, 43, 56–58), according to the accounts of most of these Australian self-trackers, their data were safe and protected: they were not being accessed and exploited by others. Even those people who expressed some misgivings about potential data breaches or leakages were not unduly concerned, as they felt that their data were not vulnerable. Indeed, many participants expressed a desire for self-tracking technologies to generate greater capacities for collecting, storing and processing even more details about them, at the same time as they were not willing or able to acknowledge the ways in which third-parties might exploit these capacities for their own benefit.

As research involving people in nations such as Canada (48) and the USA (50, 63) has found, these Australian participants viewed social privacy differently from institutional privacy. In their accounts, privacy was interpreted as relating to aspects such as how personal their details were, to what extent other people in their lives might be interested in these details and how sharing of the information might support face-to-face relationships. They felt as if they were protecting their privacy by not sharing their self-tracked data in online forums, by restricting sharing to face-to-face encounters with people they knew and trusted, or by choosing to use digital technologies to only track aspects of their lives that they did not consider to be overly revealing. The participants made distinctions about the types of personal information they were collecting in terms of which data they were willing to share with others and with whom they would share these details, similar to findings in other studies of publics' concepts of personal data privacy (48, 63). However, these participants did not tend to adopt the feelings of lacking agency or resigned acceptance toward their data privacy and security that has been identified in some other studies (50, 52). They simply either believed that their self-tracked details were secure, or that their data would be of no interest to third parties [c.f. (43, 56–58)].

In the participants' accounts, therefore, while some acknowledged the capacities of self-tracked data to contribute to close relationships, for the most part they did not acknowledge the capacities of these data to be distributed well-beyond these relationships: as lively, mobile and open to the use or misuse of other actors (11). For these people, their personal data were viewed as “locked up,” static and protected, shared only with the people they chose, in face-to-face encounters rather than very large digitally mediated networks. They were therefore quite confident that they were able to control their data privacy. While the participants did acknowledge the potential relationality of the data they generated, this was limited to situations and relationships which they specifically selected, rather than the multifarious possibilities in which their personal data could circulate in unknown data economies, viewed and exploited by unknown actors.

## Conclusion

In this article, I have shown how for self-tracked data come to matter in self-trackers' lives as part of their personal relationships. I have considered in detail how the participants' accounts of their self-tracking and data sharing practices are entangled with their concepts of data privacy and intimate relationships with their bodies and with other people. This is a more-than-digital and relational approach to privacy that emphasizes the distributed and affective nature of personal data generation and sharing. I have shown that the possibility of sharing self-tracked data is highly contextual and responsive to understandings of the role of these details to contribute to relationships. The findings highlight the continuing lack of awareness that even relatively highly educated Australians have about data mining and other third-party use of their personal data, despite regular high levels of publicity in the news media to data breaches, leaks and exploitation. As this study showed, potential data harms such as these remain vague and distant threats which have not yet had direct impacts on people like these self-trackers. They have difficulty in imagining the ways their personal data may be used by other people or agencies beyond the realm of face-to-face encounters with intimate or expert others.

This study has broader implications beyond the concepts and practices of data sharing and privacy held by self-trackers. This was a group of people who for the most part had voluntarily chosen to engage in committed and habitual self-tracking, or else had been encouraged to engage in these practices by their doctors in the interests of better managing their health. These people could identify few risks or harms to themselves of their personal data being collected and archived in ways that could possibly open access to their details by third parties. They were unaware of the extent to which their self-tracking details could be used for data profiling and other forms of data use. They felt in control of their data and viewed their self-tracking practices as beneficial to themselves, and in some cases, to others. This research could be extended by focusing attention on social groups in Australia who have been pushed or coerced into self-tracking, who are disadvantaged or socially vulnerable, who are generating health data that are potentially stigmatizing or those who have experienced privacy harms or discrimination from their personal data being accessed by third parties. Australia has a recent poor record in the misuse of personal digitized information for exacerbating socioeconomic disadvantage and exerting surveillance over already under-privileged groups (34, 64). Among other groups, Indigenous people have called for better data sovereignty, involving self-determination of what information is generated about them and better control over third-party access to their data (65). Members of such groups may have very different experiences to relate about personal data sharing and privacy. Identifying points of frictions, reinterpretations and resistances and the affective forces that can work to challenge taken-for-granted assumptions about third-party self-tracked data use could also contribute to future inquiries and theorizing about data sharing and privacy.

## Data Availability Statement

The datasets generated for this article are not readily available because participants did not provide consent for their interview data to be shared with anyone beyond the author. Requests to access the datasets should be directed to d.lupton@unsw.edu.au.

## Ethics Statement

The studies involving human participants were reviewed and approved by University of Canberra Human Research Ethics Committee. The patients/participants provided their written informed consent to participate in this study.

## Author Contributions

DL is the sole author. She planned and managed the study, analyzed the interviews, undertook all the writing of this article, and approved the final version.

## Conflict of Interest

The author declares that the research was conducted in the absence of any commercial or financial relationships that could be construed as a potential conflict of interest.
